# Nursing consultation based on supported self-care for the management of type 2 diabetes mellitus: a quasi-experimental study^*^

**DOI:** 10.1590/1518-8345.8132.4873

**Published:** 2026-07-20

**Authors:** Elen Ferraz Teston, Gabrielly Segatto Brito, Rosilene Rocha Palasson, Thaís Gianini Dias, Guilherme Oliveira de Arruda, Sonia Silva Marcon

**Affiliations:** 1 Universidade Federal de Mato Grosso do Sul, Instituto Integrado de Saúde, Campo Grande, MS, Brazil.; 2 Scholarship holder at the Coordenação de Aperfeiçoamento de Pessoal de Nível Superior (CAPES), Brazil.; 3 Universidade Estadual de Maringá, Departamento de Enfermagem, Maringá, PR, Brazil.

**Keywords:** Office Nursing, Diabetes Mellitus, Self Care, Patient Participation, Primary Health Care, Nursing

## Abstract

**Objective:**

to evaluate the effect of nursing consultations based on self-care, supported by the performance of self-care activities, on laboratory parameters in people with type 2 diabetes mellitus.

**Method:**

a quasi-experimental study was conducted with people with type 2 diabetes mellitus, with a Comparison group, whose participants received usual follow-up offered by the health unit, and an intervention group, which received three nursing consultations and two telephone monitoring calls. Data were analyzed using mixed linear models, with a significance level of 5%.

**Results:**

57 individuals participated in the study. A significant effect of the intervention was observed on adherence scores to self-care activities and on levels of total cholesterol, very low-density lipoprotein, and triglycerides.

**Conclusion:**

nursing consultations focused on supporting self-care, associated with telephone monitoring, promote understanding of the disease and engagement in the management of the chronic condition by people with type 2 diabetes mellitus, with positive effects on laboratory parameters.

## Highlights

**(1)** Increased adherence to self-care practices and improvement in laboratory parameters. **(2)** Self-care supported by primary health care: a viable, sustainable, and patient-centered practice. **(3)** Strategic role of the nurse in implementing innovative and humanized practices. **(4)** Contribution of the telephone approach to continuous care support. **(5)** Sustainable model in primary health care increases engagement and efficiency in the management of type 2 diabetes.

## Introduction

Nursing consultation (NC) demands clinical reasoning and integrates systematic assessments, individualized interventions, and educational actions aimed at promoting comprehensive care. The guidance provided during NCs is adapted to the needs and unique characteristics of patients and their families^1^, being considered a soft-hard technology, supported by structured knowledge and technical-scientific foundations that guide the nurse’s work process^2^.

Educational actions aimed at health promotion are essential, as they encourage people to reflect on their behaviors and needs for changes that favor well-being and the management of chronic conditions. Studies indicate that interventions based on supported self-care have a positive impact on the self-management of health in people with chronic conditions^3^ ^-^ ^5^, such as type 2 Diabetes Mellitus (DM2). Supported self-care is based on the 5As methodology (Assess, Advise, Agree, Assist, Arrange), which organizes the process of supporting behavior change in a structured way, guiding the professional in assessing the user’s needs, providing qualified advice, setting goals and systematic follow-up^6^.

More broadly, self-care is an essential component in promoting health and managing chronic conditions, being understood as a multidimensional process that involves knowledge, skills development and attitude change for monitoring and maintaining one’s own health condition^5^ ^,^ ^7^ ^-^ ^8^. When supported by effective communication centered on the user’s perspective, this process contributes to strengthening people’s confidence and co-responsibility in relation to their own care^8^ ^-^ ^9^.

The American Diabetes Association (ADA) strongly recommends lifestyle interventions as a central strategy for managing type 2 diabetes^10^. In this management, self-care is essential and involves activities such as frequent capillary blood glucose monitoring, healthy eating, regular physical activity, correct use of medications, and foot care^11^.

In Primary Health Care (PHC), NC based on the supported self-care framework can be a promising strategy to promote people’s engagement with their health condition. Through clinical assessment, goal setting, development of individualized care plans, and continuous follow-up, nurses can help people with type 2 diabetes to manage their health condition more effectively, using the resources available in services and in the community^1^ ^,^ ^6^ ^,^ ^12^.

Although studies point to the importance of patient-centered interventions for health promotion and self-management of the chronic condition, there is a predominance of those focused on preventing disease complications^13^ ^-^ ^15^. Therefore, gaps remain in the literature that translate the recommendations into structured nursing practices within the scope of PHC^16^. 

In this sense, highlighting that the use of a soft-hard technology such as NC can favor the proper management of DM2, can contribute to the work process in Family Health units, with the reduction of hospitalization, with adherence to treatment and self-care practices and with the improvement in quality of life^1^ ^,^ ^3^ ^,^ ^10^. Furthermore, it also contributes to the advancement of knowledge about the care offered to people with chronic conditions, especially diabetes.

Given the above, the following question arises: What are the effects of NC based on self-care supported by the adoption of self-care practices and on the laboratory parameters of people with DM2? To answer it, the objective of the study was defined as: to evaluate the effect of NC based on self-care supported by the performance of self-care activities and on laboratory parameters in people with DM2.

## Method

### Study design

This is a quasi-experimental, quantitative study linked to a matrix project funded by the Foundation for the Support of the Development of Education, Science and Technology of the State of Mato Grosso do Sul (FUNDECT), under the call for proposals of the Research Program for the SUS: shared management in health (PPSUS). The recommendations of the Transparent Reporting of Evaluations With Nonrandomized Designs (TREND)^17^ were used in the preparation of this research report.

### Location and period of data collection

The study was conducted in two Family Health Units (FHUs) in a capital city in the Brazilian Midwest. The first unit had 447 registered individuals with type 2 diabetes mellitus (DM2) and served as the comparison group; the second unit had 332 registered individuals and served as the intervention group. Data were collected between March and November 2023. 

### Population and selection criteria

The study population consisted of people with type 2 diabetes. Inclusion criteria were: being 18 years old or older, registered at one of the Family Health Units (FHU) under study, and expressing interest in participating in the research upon learning about it. Exclusion criteria were: having health conditions that would hinder or prevent verbal communication and/or travel to the unit, as reported by the Community Health Agent (CHA).

Responsibilities for discontinuation included: hospitalization during the data collection period and failure to answer three telephone contact attempts on different days and times. 

### Definition of the sample and study variables

The research was disseminated through posters displayed in the units, distribution of pamphlets, and personal invitations made by members of the Family Health team, especially the Community Health Agents (CHA). The sample was non-probabilistic, defined by convenience.

The variables of interest were those related to the characteristics of the participants (age group, gender, race, marital status, occupation, education, time since diagnosis) and the outcomes. Two specific types of outcomes were considered: a) Laboratory parameters of the tests: fasting blood glucose, total cholesterol, high-density lipoprotein (HDL), low-density lipoprotein (LDL), very low-density lipoprotein (VLDL), triglycerides, urea, creatinine, glycated hemoglobin (HbA1c); and b) Diabetes self-care activities.

### Characterization of the intervention

Individuals participating in the Comparison group received the usual follow-up from the unit to which they were linked, which includes on-demand medical consultations, distribution of medications and supplies for insulin application, and blood glucose monitoring. For the research, they underwent laboratory tests and answered a questionnaire at two different times. The members of the intervention group, in addition to the aforementioned, also received three in-person NCs and two telephone monitoring calls.

**Figure 1:**
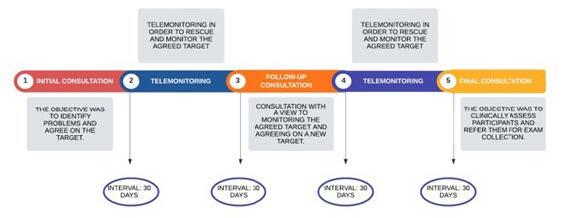
Intervention plan

The NCs were guided by an instrument, developed by the research team based on the information contained in the Self-Care manual, which is based on the “5 As” methodology (Assess, Advise, Agree, Assist, Arrange)^18^. They lasted an average of 60 minutes, were carried out according to the schedule presented in Figure 1, at the unit itself or at home, when extremely necessary - an event that prevented the patient from traveling (two cases).

In the first clinical consultation, a physical examination was performed, and perceptions about diabetes were investigated, in addition to identifying the main difficulties/problems faced in managing this condition. A clinical assessment was also carried out, consisting of blood pressure measurement, weight and height verification to determine the Body Mass Index, abdominal circumference, and capillary blood glucose measurement.

At the end of the clinical consultation, participants were asked to prioritize one of the problems/difficulties in managing type 2 diabetes and suggest actions they considered feasible to overcome it. From this, an individualized care plan was jointly developed for each participant, consisting of goals and activities to be carried out.

In the following two consultations, the clinical assessment was performed again, and the achievement of the goals was investigated. When the goals were achieved, the individual indicated another problem/difficulty and suggested actions to overcome it, establishing a new goal. If the goal was not achieved within the thirty-day period, it was discussed again, based on the slips and relapses in the behavior change process, and renegotiated, respecting the limits and possibilities of the individual.

The telemonitoring, in turn, aimed to track the agreed-upon goal, identify expectations for the next consultation, resolve doubts during the habit change process, and assist individuals with difficulties related to following the goals. For each participant, a physical record was created containing the records of information obtained during the NC and telemonitoring.

The principal researcher (a nurse, a master’s student in Nursing, duly trained to conduct the NC, develop care plans, and apply data collection instruments) had no prior relationship with the participants and was responsible for conducting all NC and telemonitoring sessions. 

### Data collection and instrument used

In the pre- and post-intervention periods, participants from both groups underwent routine laboratory tests for monitoring type 2 diabetes (DM2) [fasting blood glucose, glycated hemoglobin (HbA1c), lipid profile, urea, and creatinine]^19^ and answered the Diabetes Self-Management Activities Questionnaire (DSMQ). The test results were printed in duplicate: one copy given to the patient and the other filed in the medical record.

To assess adherence to self-care activities, the DSMQ, translated, adapted, and validated for Brazil^19^, was used. The DSMQ contains 15 items that address diet, physical activity, blood glucose monitoring, foot care, medication use, and smoking. Responses are presented on a Likert-type scale (zero to seven) corresponding to the number of days in the week the activity was performed, with zero being the worst and seven the best possible situation. The scoring is reversed in the items on fat and sweets consumption^20^. At the beginning of the research, the DMSQ was applied by the principal researcher to the members of both groups when they attended the Unit for the collection of material for laboratory tests.

### Data processing and analysis

The data were tabulated in an Excel spreadsheet. The records of the NC (Communication and Monitoring) and telemonitoring were analyzed in order to quantify the self-care goals most frequently agreed upon. Independent samples t-tests, Pearson’s chi-squared test, and Fisher’s exact test were used to compare the study groups at baseline, according to sociodemographic characteristics and time since diagnosis. The Wilcoxon test was performed for the unadjusted comparison of group scores between the two observation points.

Analysis was conducted using linear mixed models (LMM) to evaluate the effect of the intervention on self-care over time and on laboratory test values. This approach was chosen because it considers the dependence between repeated measurements performed on the same participants and because it allows the inclusion of cases with missing data in one or more observation points, under the assumption that the missing data were of the random type (Missing at Random - MAR^21^).

The pre- and post-intervention time points were specified as intra-subject factors (repeated measures effect), and the group (intervention vs. Comparison) as inter-subject factors. The “time × group” interaction effect was included to test whether the outcome variation over time differed between groups, representing the intervention effect.

The models were fitted with a random intercept per participant, allowing for the estimation of individual variability in baseline values. The “compound symmetry” covariance structure was adopted, considering the constant correlation between repeated measures (suitable for two observation time points). The analyses were adjusted for previously defined potentially confounding covariates (age, gender, race/color, and time since diagnosis), entered as fixed effects.

The assumptions of normality and homoscedasticity of the residuals were verified graphically (histogram, Q-Q plot, and graph of standardized residuals versus predicted values). Small violations were considered tolerable, given the robust nature of the mixed models. A significance level of 5% was adopted. Analyses were performed using IBM Statistical Package for the Social Sciences (SPSS)^®^ Statistics software, version 20.

### Ethical considerations

The study was developed in accordance with national and international ethical recommendations for research involving human beings, with approval from the Research Ethics Committee with Human Beings of the signatory institution (Opinion No. 4,321,389). 

## Results

**Figure 2:**
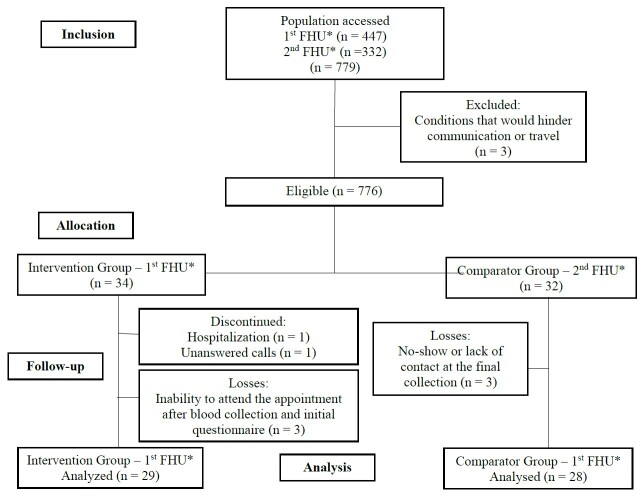
Flowchart for inclusion, allocation, follow-up, and analysis of study participants

Twenty-nine individuals were allocated to the intervention group and 28 to the Comparison group. Figure 2 shows the study flow from inclusion to data analysis.

The participants in both groups were considered statistically similar. Although the result of the proportion comparison test indicates a significant difference for education level, it is observed that the confidence intervals overlap to some extent for all observed proportions (Table 1). 

*FHU = Family Health Unit

**Table 1:** Comparison of the Intervention and Comparison groups based on sociodemographic characteristics and time since diagnosis at baseline. Campo Grande, MS, Brazil, 2023-2024

Sociodemographic variables	Intervention Group		Comparison Group		p-value
	N (%)* or Mean (SD)^‡^	CI-95%^†^	N (%)* or Mean (SD)^‡^	CI-95%^†^	
**Age (years)**	64.7 (11.3)	60.4 - 69.0	66.2 (8.64)	62.6 - 69.8	0.597^§^
**Gender**					
Female	20 (69.0)	50.6 - 82.8	20 (76.9)	57.5 - 89.2	0.508^||^
Male	9 (31.0)	17.2 - 49.4	6 (23.1)	10.8 - 42.5	
**Race**					
White	15 (51.7)	34.4 - 68.6	12 (46.2)	28.8 - 64.5	0.187^¶^
Brown	6 (20.7)	9.6 - 38.9	10 (38.5)	22.4 - 57.5	
Black	8 (27.6)	14.6 - 46.0	3 (11.5)	3.33 - 30.0	
Yellow	0 (0.0)	0.00 - 0.00	1 (3.8)	0.00 - 20.7	
**Marital status**					
Married	18 (62.1)	43.9 - 77.3	11 (44.0)	26.7 - 62.9	0.342^¶^
Single	2 (6.9)	0.09 - 23.3	5 (20.0)	8.5 - 39.7	
Separeted	2 (6.9)	0.09 - 23.3	0 (0.0)	0.00 - 16.1	
Divorced	3 (10.3)	2.9 - 27.4	4 (16.0)	5.9 - 35.4	
Widow	4 (13.8)	5.0 - 31.3	5 (20.0)	8.5 - 39.7	
**Occupation**					
Retired	13 (44.8)	28.4 - 62.4	11 (42.3)	25.6 - 61.1	0.262^||^
No formal occupation	6 (20.7)	9.6 - 38.9	10 (38.5)	22.4 - 57.5	
Others	10 (34.5)	19.9 - 52.8	5 (19.2)	8.2 - 38.5	
**Education**					
Illiterate	2 (6.9)	0.09 - 23.3	1 (3.8)	0.00 - 20.7	0.031^¶^
Incomplete Elementary Education	12 (41.4)	25.5 - 59.3	20 (76.9)	57.5 - 89.2	
Completed elementary education	9 (31.0)	17.2 - 49.4	1 (3.8)	0.00 - 20.7	
Incomplete High School	1 (3.4)	0.00 - 18.9	1 (3.8)	0.00 - 20.7	
Completed High School	4 (13.8)	5.0 - 31.3	3 (11.5)	3.3 - 30.0	
Completed higher education	1 (3.4)	0.00 - 18.9	0 (0.0)	0.00 - 15.6	
**Time to diagnosis (years)**	7.31 (7.58)	3.9 - 10.6	10.3 (10.3)	6.6 - 13.9	0.234^§^

*N (%) = Absolute number and percentage; ^†^CI-95% = Confidence interval for the proportion; ^‡^SD = Standard deviation; ^§^t-test for independent samples; ^||^X^2^ from Pearson; ^¶^ Fisher’s Exact Test

The goal most frequently agreed upon during the NC was healthy eating (65.52%), with an emphasis on reducing fats and carbohydrates and increasing fruit consumption. The practice of physical activity, mainly walking three times a week for 30 minutes, was the second most agreed upon goal (58.62%), followed by increased water intake (37.93%) and stress management through enjoyable activities (27.59%).

According to Table 2, the self-care activity scores showed a significant difference between the pre- and post-intervention periods for the intervention group, while for the Comparison group the difference was not statistically significant.

**Table 2:** Comparison of Diabetes Self-Management Activities scores in the Intervention Group and the Comparison Group before and after the intervention. Campo Grande, MS, Brazil, 2023-2024

Scores	Moments						p-value*
	Pre intervention			Post intervention			
	Mean	Median	Standard Deviation	Mean	Median	Standard Deviation	
Intervention Group DSMQ^†^	3.57	3.43	0.82	4.29	4.43	0.76	<0.001
Comparison Group DSMQ^†^	3.65	3.57	0.92	3.98	3.72	1.20	0.084

*Wilcoxon`s test; ^†^DSMQ = Diabetes Self-Management Activities Questionnaire

The linear mixed model indicated a significant interaction effect of “time x group” on the outcome (F (50.0)=5.30; p=0.025), demonstrating that the intervention produced a significantly different effect from the result obtained by the Comparison group. The superior effect of the intervention is evident when analyzing the coefficient estimated and adjusted for the variables gender, age, race/color, and time since diagnosis, which was equal to 0.55 (standard error = 0.23; 95% CI = 0.07 - 1.03). Therefore, the positive variation in the diabetes self-management score values among people who received the intervention was significantly different from the variation in the Comparison group over time.

Table 3 shows that, according to the linear mixed models, there was a significant positive effect of the intervention on the values of Total Cholesterol, VLDL, and Triglycerides. This shows that the variation between pre- and post-intervention time points was different between the groups regardless of the covariates analyzed.

In proportional terms and taking into account the estimated coefficient values contained in Table 3 (which discounts the effect on the Comparison group), the reductions in Fasting Blood Glucose, Total Cholesterol and Triglycerides for the Intervention Group stand out, being, respectively, 16.7% (mean = 171.41 mg/dl), 11.7% (mean = 199.52 mg/dl) and 32% (mean = 228.55 mg/dl).

**Table 3:** Effects of the intervention based on the “time x group” interaction on laboratory test values. Campo Grande, MS, Brazil, 2023-2024

Variable	Coefficient (β)*	Standard Error	CI95%^†^ Inferior	CI95%^†^ Superior	p-value^‡^
Fasting Blood Glucose	- 28.59	16.44	61.62	4.43	0.088
Total Cholesterol	23.23	8.86	41.05	5.42	**0.012**
HDL^‡^	0.12	1.89	3.93	3.69	0.949
LDL^§^	0.08	6.74	13.47	13.65	0.990
VLDL^||^	8.74	2.80	14.37	3.11	**0.003**
Triglycerides	73.18	26.84	127.11	19.26	**0.009**
Urea	4.10	2.57	9.27	1.07	0.118
Creatinine	0.02	0.03	0.09	0.04	0.480
HbA1c^¶^	0.57	0.37	1.31	0.17	0.130

*β = Estimated coefficient, adjusted for the variables sex, age, race/color, and time since diagnosis; ^†^CI95% = 95% confidence interval; ^‡^Mixed linear models; ^‡^HDL = High-density lipoprotein; ^§^LDL = Low-density lipoprotein; ^||^VLDL = Very low-density lipoprotein; ^¶^HbA1c = Glycated Hemoglobin. The interaction term Momentum×Group represents the differential effect of the intervention between the groups over time.

## Discussion

The results of this study demonstrated positive effects of NC based on supported self-care on self-care practices and levels of total cholesterol, very low-density lipoprotein, and triglycerides in people with type 2 diabetes mellitus (DM2) followed up in primary health care. This result indicates that non-pharmacological interventions conducted by nurses can contribute to the management of the disease and prevention of cardiovascular complications, reinforcing the findings of previous research on the effectiveness of strategies to encourage self-care in people with DM2^22^ ^-^ ^24^.

The individualized approach, with the agreement of realistic goals and continuous monitoring, favored the active involvement of participants in care. This collaborative process, based on listening and co-responsibility, encourages the protagonism and self-confidence of people with DM2, corroborating studies that highlight the role of the nurse as a facilitator of sustainable behavioral changes^3^ ^,^ ^9^ ^,^ ^25^.

Telemonitoring proved to be an important support resource between consultations, enabling the reinforcement of guidelines, the review of goals, and the clarification of doubts. This type of follow-up expands access, strengthens the therapeutic bond, and sustains self-care behaviors over time. These benefits were also described in a study that used a mobile health application as a form of intervention^26^.

In addition to the clinical and behavioral benefits, the findings of the present study reinforce the relevance of patient-centered care with appreciation for the social, cultural, and emotional context of each person. Thus, the use of accessible language, the shared construction of the care plan, and the encouragement of autonomy are strategies that make the educational process more meaningful and effective^27^ ^-^ ^28^.

The significant reduction in total cholesterol, VLDL, and triglyceride levels in the participants of the intervention group may be related to the main goals agreed upon during the NC, which were improvements in eating habits and physical activity. This evolution reflects the positive impact of the intervention on the knowledge, autonomy, and engagement of the participants, central aspects in the management of chronic conditions^9^ ^,^ ^23^. 

However, these results differ from a systematic review of 18 studies conducted in Africa, totaling 2,599 participants, which did not show statistically significant improvements in the lipid profile after interventions aimed at self-management of diabetes^29^. The authors attributed these findings to limitations regarding the heterogeneity of the interventions, as well as the need for trained professionals, adequate resources and attention to health literacy for people with type 2 diabetes.

From a clinical management perspective, the effect observed on total cholesterol differs from a study conducted in Mexico City with 2,334 people, in which a slight increase in total cholesterol was observed as an effect of the application of a multimedia instrument^30^. Regarding the effect on triglycerides, the same study found a reduction of 7.6% (from 227.78 to 210.38 mg/dl; p = 0.001), which indicates that the integration between strategies (nursing consultation with telemonitoring) may favor the achievement of better results.

The standard error of the estimated coefficient for fasting blood glucose was considered high (57.5% of the coefficient value), which may be directly related to the fact that the difference between the groups was not considered statistically significant. However, the observed effect on fasting blood glucose is comparable to effects observed in the literature and which were also considered statistically significant^31^.

In an intervention carried out by nurses, based on nursing consultations and a care plan, with 249 people with type 2 diabetes in Spain, a 10.1% reduction in glycemic values (based on HbA1c) was found^31^. In a study carried out in Thailand, the adjusted effect observed was a 7.3% reduction in glycemic values in 77 people with type 2 diabetes, from a 4-month intervention based on education for diabetes self-management^32^.

The incorporation of self-care-based practices supported in primary health care is feasible and consistent with the principles of comprehensiveness and continuity of care. However, the professional’s willingness is not enough; it requires investment in professional training, reorganization of work processes, and institutional support to ensure the sustainability of actions and integration between different levels of care^33^. A study conducted in southern Brazil found that although nurses believe that the use of technologies can favor communication with other points in the RAS and with the patient, optimizing and supporting teamwork, they consider that the absence of training and institutional support, and the insufficiency of equipment and human resources are factors that affect and may make the use of telemonitoring unfeasible in the follow-up of people with chronic conditions such as hypertension and diabetes in PHC^34^.

The reorganization of work processes is a requirement for practices that endorse the engagement of users with chronic conditions to take place within the scope proposed in the Chronic Conditions Care Model. The implementation of public health policy faces obstacles in the micro-scenario of health care, and in the case of care for chronic conditions, this involves: divergences between the fulfillment of individual tasks by professionals and collective involvement, predominance of curative care, lack of planning and professional integration, and work pressure related to goals and deadlines based on productivity^35^.

It is observed in the literature that the provision of technical-pedagogical support to primary health care professionals is part of the process of reorganizing care and that it has contributed to the implementation of soft and semi-hard technologies within this level of care and, consequently, to the stabilization of the health status of users with chronic conditions^36^. It is therefore highlighted that for the practical application of supported self-care in primary health care, the initial step must involve management, continuing education sectors and universities in supporting professionals at the point of health care, so that they feel supported and involved by the culture of professional-user co-responsibility.

Although the present study demonstrates that the use of nurse-supported self-care principles produces positive effects on clinical outcomes, it is understood that its applicability goes beyond uniprofessional practice. For the national context, the involvement of Community Health Agents (CHAs) is a fundamental condition when considering the implementation and support of engagement, counseling, and telemonitoring practices for such conditions^37^. The role of physicians, physical education professionals, nutritionists, and psychologists should be considered according to the local and regional specificities of the Health Care Network and the needs and goals of the users. Providing opportunities for individual and group-based shared care modalities can broaden the reach of nurse-supported self-care in practice^24^ ^-^ ^25^. 

Among the possible limitations of this study, the absence of risk stratification stands out, as well as external factors, such as failures in medication distribution during the data collection period, which were not controlled in the data analysis. Another aspect to be considered was the difficulty in obtaining the active involvement of primary health care nurses in the clinical trials as initially agreed, since the proposal aimed at applicability within the Brazilian Unified Health System (SUS). The justifications presented for the low participation included the overloaded daily routine of primary care and the perception that the presence of the principal researcher in conducting the activities would eliminate the need for direct participation of other professionals. In any case, the results offer support for strengthening nursing as a scientific and social practice, highlighting the potential of clinical trials based on supported self-care to promote the autonomy of individuals in managing diabetes and improve clinical outcomes.

## Conclusion

Nursing consultations based on supported self-management have demonstrated a positive effect on engagement in self-care practices in people with type 2 diabetes mellitus (DM2) followed up PHC and on laboratory parameters, especially total cholesterol, very low-density lipoprotein, and triglycerides. These results show that structured educational interventions, centered on goal setting and accompanied by continuous support, can promote engagement, autonomy, and better management of the chronic condition.

The findings reinforce the strategic role of nurses in implementing innovative and humanized care practices capable of integrating clinical, educational, and relational aspects. The systematic adoption of nursing consultations based on supported self-care contributes to the consolidation of a patient-centered care model aligned with the principles of comprehensiveness and co-responsibility.

It is recommended that future studies expand intervention strategies, including the analysis of the effects of multidisciplinary action, collective approaches, digital technologies, and different care contexts. This expansion of approaches may strengthen the evidence on the effectiveness and applicability of supported self-care in addressing chronic conditions.

## Data Availability

Datasets related to this article will be available upon request to the corresponding author.
