# Supplementary material for: Nursing consultation based on supported self-care for the management of type 2 diabetes mellitus: a quasi-experimental study
Source: Rev Lat Am Enfermagem. 2026 Jul 20;34:e4873. doi: 10.1590/1518-8345.8132.4873 (PMC13387792; doi:10.1590/1518-8345.8132.4873)
Supplement: Translation

# Translation: Consulta de Enfermagem baseada no autocuidado apoiado para manejo do diabetes mellitus tipo 2: estudo quase experimental^*^

**DOI:** 10.1590/1518-8345.8132.4874

**Authors:** Elen Ferraz Teston, Gabrielly Segatto Brito, Rosilene Rocha Palasson, Thaís Gianini Dias, Guilherme Oliveira de Arruda, Sonia Silva Marcon

**Affiliations:** 1 Universidade Federal de Mato Grosso do Sul, Instituto Integrado de Saúde, Campo Grande, MS, Brasil.; 2 Scholarship holder at the Coordenação de Aperfeiçoamento de Pessoal de Nível Superior (CAPES), Brasil.; 3 Universidade Estadual de Maringá, Departamento de Enfermagem, Maringá, PR, Brasil.

**Keywords:** Enfermagem Ambulatorial, Diabetes Mellitus, Autocuidado, Engajamento do Paciente, Atenção Primária à Saúde, Enfermagem.

## Abstract

**Objetivo:**

avaliar o efeito da consulta de enfermagem baseada no autocuidado apoiado na realização de atividades de autocuidado e sobre parâmetros laboratoriais em pessoas com diabetes mellitus tipo 2.

**Método:**

estudo quase experimental junto a pessoas com diabetes mellitus tipo 2, com grupo comparador, cujos participantes receberam acompanhamento habitual oferecido pela unidade, e grupo intervenção, que recebeu três consultas de enfermagem e duas ligações telefônicas de monitoramento. Dados analisados mediante modelos lineares mistos, com significância de 5%.

**Resultados:**

participaram do estudo 57 indivíduos. Observou-se efeito significativo da intervenção sobre os escores de adesão às atividades de autocuidado e nos níveis de colesterol total, lipoproteína de muito baixa densidade, triglicérides.

**Conclusão:**

a consulta de enfermagem centrada no apoio ao autocuidado associada ao monitoramento telefônico, favorece a compreensão sobre a doença e o engajamento no manejo da condição crônica por pessoas com diabetes mellitus tipo 2, com reflexos nos parâmetros laboratoriais.

## Destaques

**(1)** Aumento na adesão às práticas de autocuidado e melhora nos parâmetros laboratoriais. **(2)** Autocuidado apoiado na APS: prática viável, sustentável e centrada no paciente. **(3)** Papel estratégico do enfermeiro na implementação de práticas inovadoras e humanizadas. **(4)** Contribuição da abordagem telefônica no suporte contínuo ao cuidado. **(5)** Modelo sustentável na APS amplia engajamento e eficiência no manejo do DM2.

## Introdução

A consulta de enfermagem (CE) demanda raciocínio clínico e integra avaliações sistemáticas, intervenções individualizadas e ações educativas que visam à promoção do cuidado integral. As orientações realizadas durante as CE são adaptadas às necessidades e singularidades dos usuários e de suas famílias^1^ sendo considerada uma tecnologia leve-dura, sustentada por saberes estruturados e fundamentos técnicos-científicos que norteiam o processo de trabalho do enfermeiro^2^. 

As ações educativas voltadas à promoção da saúde são essenciais, pois estimulam as pessoas a refletirem sobre comportamentos e necessidades de mudanças que favoreçam o bem-estar e o manejo de condições crônicas. Estudos indicam que intervenções baseadas no autocuidado apoiado exercem impacto positivo na autogestão da saúde de pessoas com condição crônica^3^ ^-^ ^5^, como o Diabetes Mellitus tipo 2 (DM2). O autocuidado apoiado fundamenta-se na metodologia dos 5As (Avaliar, Aconselhar, Acordar, Assistir e Acompanhar), que organiza de forma estruturada o processo de apoio à mudança de comportamento, orientando o profissional na avaliação das necessidades do usuário, no aconselhamento qualificado, na pactuação de metas e no acompanhamento sistemático^6^.

De forma mais ampla, o autocuidado constitui um componente essencial na promoção da saúde e no manejo das condições crônicas, sendo compreendido como um processo multidimensional que envolve conhecimento, desenvolvimento de habilidades e mudança de atitude para o monitoramento e manutenção da própria condição de saúde^5^ ^,^ ^7^ ^-^ ^8^. Quando apoiado em uma comunicação efetiva e centrada na perspectiva do usuário, esse processo contribui para o fortalecimento da confiança e da corresponsabilização das pessoas em relação ao seu próprio cuidado^8^ ^-^ ^9^.

A *American Diabetes Association* (ADA) recomenda fortemente intervenções no estilo de vida como estratégia central para o manejo do DM2^10^. Nesse manejo, o autocuidado é essencial e envolve atividades como o monitoramento frequente da glicemia capilar, alimentação saudável, prática de atividade física regular, uso correto das medicações e cuidados com os pés^11^. 

Na Atenção Primária à Saúde (APS), a CE fundamentada no referencial do autocuidado apoiado pode constituir uma estratégia promissora para promover o engajamento das pessoas em relação à sua condição de saúde. Por meio da avaliação clínica, pactuação de metas, elaboração de planos de cuidado individualizados e acompanhamento contínuo, o enfermeiro pode auxiliar as pessoas com DM2 a realizarem o manejo mais eficaz de sua condição de saúde, utilizando os recursos disponíveis nos serviços e na comunidade^1^ ^,^ ^6^ ^,^ ^12^. 

Embora estudos apontem a importância das intervenções centradas no paciente para a promoção da saúde e autogestão da condição crônica, constata-se a predominância daqueles focados na prevenção de complicações da doença^13^ ^-^ ^15^. Portanto, persistem lacunas na literatura que traduzam as recomendações em práticas estruturadas de enfermagem no âmbito da APS^16^. 

Nesse sentido, evidenciar que o uso de uma tecnologia leve-dura como a CE pode favorecer o manejo adequado do DM2 viabiliza contribuições com o processo de trabalho nas unidades de Saúde da Família, com a redução de hospitalização, com a adesão ao tratamento e às práticas de autocuidado e com a melhora na qualidade de vida^1^ ^,^ ^3^ ^,^ ^10^. Para além, contribui também com o avanço do conhecimento sobre a assistência oferecida às pessoas com condições crônicas, especialmente o diabetes.

Diante do exposto, surge o seguinte questionamento: quais são os efeitos da CE baseada no autocuidado apoiado na adoção de práticas de autocuidado e sobre os parâmetros laboratoriais de pessoas com DM2? Para respondê-lo, definiu-se como objetivo do estudo: avaliar o efeito da CE baseada no autocuidado apoiado na realização de atividade de autocuidado e sobre parâmetros laboratoriais em pessoas com DM2.

## Método

### Delineamento do estudo

Estudo quase experimental, de natureza quantitativa, vinculado a um projeto matricial financiado pela Fundação de Apoio ao Desenvolvimento do Ensino, Ciência e Tecnologia do Estado de Mato Grosso do Sul (FUNDECT), no edital do Programa Pesquisa para o SUS: gestão compartilhada em saúde (PPSUS). As recomendações do *Transparent Reporting of Evaluations With Nonrandomized Designs* (TREND)^17^ foram utilizadas na elaboração desse relatório de pesquisa.

### Local e período da coleta de dados

O estudo foi realizado em duas Unidades de Saúde da Família (USFs) em uma capital do Centro-Oeste brasileiro. A primeira tinha 447 pessoas com DM2 cadastradas, e foi destinada para o grupo comparador; a segunda tinha 332 pessoas cadastradas e foi destinada ao grupo intervenção. Os dados foram coletados no período de março a novembro de 2023. 

### População e critérios de seleção

A população do estudo foi composta por pessoas com DM2. Foram critérios de inclusão: 18 anos ou mais, cadastrado em uma das USF em estudo e manifestação de interesse por participar da pesquisa ao ter conhecimento sobre ela. Os critérios de exclusão foram: apresentar condições de saúde que dificultariam ou impossibilitariam a comunicação verbal e/ou o deslocamento até a unidade, conforme informado pelo ACS.

Considerou-se como motivos de descontinuidade: hospitalização no período de coleta de dados e o não atendimento de três tentativas de contato telefônico em dias e horários distintos. 

### Definição da amostra e variáveis de estudo

A pesquisa foi divulgada mediante cartazes fixados nas unidades, distribuição de panfletos e convites pessoais realizados pelos membros da equipe de Saúde da Família, sobretudo os Agentes Comunitários de Saúde (ACS). A amostra foi do tipo não probabilística, definida por conveniência. 

As variáveis de interesse foram aquelas relacionadas com a caracterização dos participantes (faixa etária, sexo, raça, estado civil, ocupação, escolaridade, tempo de diagnóstico) e com os desfechos. Foram considerados dois tipos específicos de desfechos: a) Parâmetros laboratoriais dos exames: glicemia de jejum, colesterol total, lipoproteína de alta densidade (HDL), lipoproteína de baixa densidade (LDL), lipoproteína de muito baixa densidade (VLDL), triglicérides, ureia, creatinina, hemoglobina glicada (HbA1c); e b) Atividades de autocuidado com o diabetes.

### Caracterização da intervenção

Os indivíduos participantes do grupo comparador receberam o acompanhamento habitual da unidade em que estavam vinculados, o que inclui consultas médicas por demanda, distribuição de medicamentos e de insumos para aplicação de insulina e monitoramento da glicemia. Para a pesquisa, realizou-se exames laboratoriais e os participantes responderam ao questionário em dois momentos. Já os integrantes do grupo intervenção, além do já citado, receberam também três CE presenciais e duas ligações telefônicas de monitoramento.

As CE foram guiadas por um instrumento elaborado pela equipe de pesquisa com base nas informações contidas no manual do Autocuidado, o qual é fundamentado na metodologia dos “5 As” (Avaliar, Aconselhar, Acordar, Assistir e Acompanhar)^18^. Com duração média de 60 minutos, as etapas foram realizadas conforme cronograma apresentado na Figura 1, na própria unidade ou no domicílio, quando extremamente necessário - intercorrência que impossibilitou o deslocamento do paciente (dois casos). 

**Figura 1:** Esquema de intervenção

Na primeira CE, realizou-se o exame físico e investigou-se a percepção sobre o diabetes, além de se identificar as principais dificuldades/problemas enfrentados para o manejo dessa condição. Também foi feita uma avaliação clínica composta por aferição da pressão arterial, verificação do peso e altura para determinação do Índice de Massa Corporal, circunferência abdominal e medição da glicemia capilar.

Ao final da CE foi solicitado que os participantes priorizassem um dos problemas/dificuldades no manejo do DM2 e sugerissem ações que consideravam viáveis para contorná-lo. A partir disto, foi elaborado, de forma conjunta, um plano de cuidados individualizado para cada participante, constituído por metas e atividades a serem realizadas.

Nas duas consultas seguintes, a avaliação clínica foi realizada novamente e investigado o cumprimento das metas. Quando as metas eram alcançadas, o indivíduo indicava outro problema/dificuldade e sugeria ações para contorná-lo(a), estabelecendo uma nova meta. Se a meta não fosse alcançada no período de trinta dias, ela era discutida novamente, com base nos deslizes e recaídas do processo de mudança de comportamento, e repactuada, respeitando os limites e as possibilidades do indivíduo.

O telemonitoramento, por sua vez, teve por objetivo acompanhar a meta pactuada, identificar as expectativas para a próxima consulta, sanar dúvidas durante o processo de mudança de hábito e auxiliar os indivíduos nas dificuldades relacionadas ao seguimento das metas. Para cada participante, criou-se um prontuário físico contendo os registros das informações obtidas durante as CE e os telemonitoramentos.

A pesquisadora principal (enfermeira, mestranda em Enfermagem, devidamente treinada para a realização da CE, elaboração de planos de cuidados e aplicação dos instrumentos de coleta de dados) não tinha qualquer tipo de relação anterior com os participantes), foi responsável pela realização de todas as CE e telemonitoramentos. 

### Coleta de dados e instrumento utilizado

Nos períodos pré e pós-intervenção, os participantes de ambos os grupos realizaram exames laboratoriais de rotina para o acompanhamento do DM2 [glicemia jejum, hemoglobina glicada (HbA1c), lipidograma, ureia e creatinina]^19^ e responderam ao Questionário de Atividades de Autocuidado com o Diabetes (QAD). Os resultados dos exames foram impressos em duas vias: uma entregue ao paciente e outra arquivada no prontuário.

Para avaliar a adesão às atividades de autocuidado, utilizou-se o QAD, traduzido, adaptado e validado para o Brasil^19^. O QAD contém 15 itens que abordam alimentação, atividade física, monitoramento da glicemia, cuidado com os pés, uso de medicação e tabagismo. As respostas são apresentadas em escala do tipo Likert (zero a sete) correspondendo ao número de dias na semana em que a atividade foi realizada, sendo zero a pior e sete a melhor situação possível. A pontuação é invertida nos itens sobre o consumo de gordura e doces^20^. No início da pesquisa o QAD foi aplicado pela pesquisadora principal junto aos integrantes dos dois grupos, quando os mesmos compareciam à Unidade para coleta de material para os exames laboratoriais. 

### Tratamento e análise dos dados

Os dados foram tabulados em uma planilha por meio do *software* Excel. Os registros das CE e telemonitoramentos foram analisados com o intuito de quantificar as metas de autocuidado mais frequentemente pactuadas. Os testes t para amostras independentes, X^2^ de Pearson e Exato de Fisher foram empregados para comparação dos grupos de estudo na linha de base, conforme características socidemográficas e tempo de diagnóstico. Realizou-se o teste de Wilcoxon para a comparação não ajustada dos escores dos grupos entre os dois momentos de observação. 

Conduziu-se análise por meio de modelos lineares mistos (*Linear Mixed Models* - LMM) para avaliar o efeito da intervenção sobre o autocuidado ao longo do tempo e sobre os valores de exames laboratoriais. Essa abordagem foi escolhida por considerar a dependência entre as medidas repetidas realizadas nos mesmos participantes e por permitir a inclusão de casos com dados ausentes em um ou mais momentos de observação, sob a suposição de que os dados faltantes eram do tipo aleatório (*Missing at Random* - MAR)^21^.

O momento pré e pós-intervenção foi especificado como fator intrassujeito (efeito de medidas repetidas) e o grupo (intervenção vs comparador) como fator intersujeitos. O efeito de interação “momento × grupo” foi incluído para testar se a variação do desfecho ao longo do tempo diferiu entre os grupos, representando o efeito da intervenção**.**

Os modelos foram ajustados com intercepto aleatório por participante**,** permitindo estimar a variabilidade individual nos valores de linha de base. A estrutura de covariância “*compound symmetry*” foi adotada, considerando a correlação constante entre as medidas repetidas (adequada para dois momentos de observação). As análises foram ajustadas por covariáveis potencialmente confundidoras previamente definidas (idade, sexo, raça/cor e tempo de diagnóstico), inseridas como efeitos fixos. 

Os pressupostos de normalidade e homocedasticidade dos resíduos foram verificados graficamente (histograma, *Q-Q plot* e gráfico de resíduos padronizados versus valores preditos). Pequenas violações foram consideradas toleráveis, dado o caráter robusto dos modelos mistos. Adotou-se o nível de significância de 5%. As análises foram realizadas no *software* IBM *Statistical Package for the Social Sciences* (SPSS)^®^ *Statistics*, versão 20.

### Considerações éticas

O estudo foi desenvolvido em conformidade com as recomendações éticas nacionais e internacionais para pesquisas envolvendo seres humanos, com aprovação pelo Comitê de Ética em Pesquisa com Seres Humanos da instituição signatária (Parecer nº 4.321.389). 

## Resultados

Foram alocados 29 indivíduos no grupo intervenção e 28 no grupo comparador. A Figura 2 apresenta o fluxo do estudo da inclusão à análise dos dados.

**Figura 2:** Fluxograma de inclusão, alocação, seguimento e análise dos participantes do estudo

*USF = Unidade de Saúde da Família

Os participantes de ambos os grupos foram considerados semelhantes do ponto de vista estatístico. Embora o resultado do teste de comparação de proporções aponte diferença significativa para escolaridade, observa-se que os intervalos de confiança se sobrepõem em alguma medida para todas as proporções observadas (Tabela 1). 

**Tabela 1:** Comparação dos grupos Intervenção e Comparador com base em características sociodemográficas e tempo de diagnóstico na linha de base. Campo Grande, MS, Brasil, 2023- 2024

Variáveis sociodemográficas	Grupo Intervenção		Grupo Comparador		p-valor
	N (%)* ou Média (Dp)^‡^	IC-95%^†^	N (%)* ou Média (Dp)^‡^	IC-95%^†^	
**Idade (anos)**	64,7 (11,3)	60,4 - 69,0	66,2 (8,64)	62,6 - 69,8	0,597^§^
**Sexo**					
Feminino	20 (69,0)	50,6 - 82,8	20 (76,9)	57,5 - 89,2	0,508^||^
Masculino	9 (31,0)	17,2 - 49,4	6 (23,1)	10,8 - 42,5	
**Raça**					
Branco	15 (51,7)	34,4 - 68,6	12 (46,2)	28,8 - 64,5	0,187^¶^
Pardo	6 (20,7)	9,6 - 38,9	10 (38,5)	22,4 - 57,5	
Preto	8 (27,6)	14,6 - 46,0	3 (11,5)	3,33 - 30,0	
Amarelo	0 (0,0)	0,00 - 0,00	1 (3,8)	0,00 - 20,7	
**Estado civil**					
Casado	18 (62,1)	43,9 - 77,3	11 (44,0)	26,7 - 62,9	0,342^¶^
Solteiro	2 (6,9)	0,09 - 23,3	5 (20,0)	8,5 - 39,7	
Desquitado	2 (6,9)	0,09 - 23,3	0 (0,0)	0,00 - 16,1	
Divorciado	3 (10,3)	2,9 - 27,4	4 (16,0)	5,9 - 35,4	
Viúvo	4 (13,8)	5,0 - 31,3	5 (20,0)	8,5 - 39,7	
**Ocupação**					
Aposentado	13 (44,8)	28,4 - 62,4	11 (42,3)	25,6 - 61,1	0,262^||^
Do lar	6 (20,7)	9,6 - 38,9	10 (38,5)	22,4 - 57,5	
Outros	10 (34,5)	19,9 - 52,8	5 (19,2)	8,2 - 38,5	
**Escolaridade**					
Analfabeto	2 (6,9)	0,09 - 23,3	1 (3,8)	0,00 - 20,7	0,031^¶^
Ens. Fundamental Incompleto	12 (41,4)	25,5 - 59,3	20 (76,9)	57,5 - 89,2	
Ens. Fundamental Completo	9 (31,0)	17,2 - 49,4	1 (3,8)	0,00 - 20,7	
Ens. Médio Incompleto	1 (3,4)	0,00 - 18,9	1 (3,8)	0,00 - 20,7	
Ens. Médio Completo	4 (13,8)	5,0 - 31,3	3 (11,5)	3,3 - 30,0	
Ensino Superior completo	1 (3,4)	0,00 - 18,9	0 (0,0)	0,00 - 15,6	
**Tempo de diagnóstico (anos)**	7,31 (7,58)	3,9 - 10,6	10,3 (10,3)	6,6 - 13,9	0,234^§^

*N (%) = Número absoluto e percentual; ^†^IC-95% = Intervalo de confiança para a proporção; ^‡^Dp = Desvio-Padrão; ^§^Teste t para amostras independentes; ^||^X^2^ de Pearson; ^¶^Teste Exato de Fisher

A meta pactuada com maior frequência durante as CE foi a alimentação saudável (65,52%), com ênfase na redução de gorduras e carboidratos e maior consumo de frutas. A prática de atividade física, principalmente no que se refere a caminhadas três vezes por semana, por 30 minutos, foi a segunda meta mais pactuada (58,62%), seguida pelo aumento na ingestão de água (37,93%) e o manejo do estresse por meio de atividades prazerosas (27,59%).

De acordo com a Tabela 2, os escores de atividades de autocuidado apresentaram diferença significativa entre os momentos pré e pós para o grupo intervenção, ao passo que, para o grupo comparador a diferença não foi estatisticamente significativa.

**Tabela 2:** Comparação dos escores de Atividades de Autocuidado com o Diabetes no Grupo Intervenção e Grupo Comparador nos momentos pré e pós-intervenção. Campo Grande, MS, Brasil, 2023-2024

Escores	Momentos						p-valor*
	Pré-intervenção			Pós-intervenção			
	Média	Mediana	Desvio-padrão	Média	Mediana	Desvio-padrão	
Grupo Intervenção QAD^†^	3,57	3,43	0,82	4,29	4,43	0,76	<0,001
Grupo Comparador QAD^†^	3,65	3,57	0,92	3,98	3,72	1,20	0,084

*Teste de Wilcoxon; ^†^QAD = Questionário de Atividades de Autocuidado com o Diabetes

O modelo linear misto indicou efeito significativo da interação “momento x grupo” sobre o desfecho (F (50,0)=5,30; p=0,025), demonstrando que a intervenção produziu efeito significativamente distinto do resultado obtido pelo grupo comparador. O efeito superior da intervenção fica evidente ao se analisar o coeficiente estimado e ajustado pelas variáveis sexo, idade, raça/cor e tempo de diagnóstico, que foi igual a 0,55 (erro-padrão = 0,23; IC95% = 0,07 - 1,03). Portanto, a variação positiva nos valores do escore de autocuidado com o diabetes entre as pessoas que receberam a intervenção foi significativamente diferente da variação no grupo comparador ao longo do tempo. 

A Tabela 3 mostra que, de acordo com os modelos lineares mistos, houve efeito positivo significativo da intervenção sobre os valores de Colesterol Total, VLDL e Triglicérides. Isto evidencia que a variação entre momentos pré e pós-intervenção foi diferente entre os grupos independentemente das covariáveis analisadas.

Em termos proporcionais e levando-se em consideração os valores do coeficiente estimado contidos na Tabela 3 (que desconta o efeito sobre o grupo comparador), destacam-se as reduções nos valores de Glicemia de Jejum, Colesterol Total e Triglicérides para o Grupo Intervenção que foram, respectivamente, de 16,7% (média = 171,41 mg/dl), 11,7% (média = 199,52 mg/dl) e 32% (média = 228,55 mg/dl).

**Tabela 3:** Efeitos da intervenção com base na interação “momento x grupo” sobre os valores de exames laboratoriais. Campo Grande, MS, Brasil, 2023-2024

Variável	Coeficiente (β)*	Erro-Padrão	IC95%^†^ Inferior	IC95%^†^ Superior	p-valor^‡^
Glicemia Jejum	- 28,59	16,44	61,62	4,43	0,088
Colesterol Total	23,23	8,86	41,05	5,42	**0,012**
HDL^‡^	0,12	1,89	3,93	3,69	0,949
LDL^§^	0,08	6,74	13,47	13,65	0,990
VLDL^||^	8,74	2,80	14,37	3,11	**0,003**
Triglicérides	73,18	26,84	127,11	19,26	**0,009**
Ureia	4,10	2,57	9,27	1,07	0,118
Creatinina	0,02	0,03	0,09	0,04	0,480
HbA1c^¶^	0,57	0,37	1,31	0,17	0,130

*β = Coeficiente estimado, ajustado pelas variáveis sexo, idade, raça/cor e tempo de diagnóstico; ^†^IC95% = Intervalo de confiança de 95%; ^‡^Modelos lineares mistos; ^‡^HDL = Lipoproteína de alta densidade; ^§^LDL = Lipoproteína de baixa densidade; ^||^VLDL = Lipoproteína de muito baixa densidade; ^¶^HbA1c = Hemoglobina Glicada. O termo de interação Momento×Grupo representa o efeito diferencial da intervenção entre os grupos ao longo do tempo

## Discussão

Os resultados deste estudo demonstraram efeitos positivos da CE baseada no autocuidado apoiado sobre as práticas de autocuidado e sobre os níveis de colesterol total, lipoproteína de muito baixa densidade, triglicérides de pessoas com DM2 acompanhadas na APS. Esse resultado indica que intervenções não farmacológicas conduzidas por enfermeiros podem contribuir para o manejo da doença e prevenção de complicações cardiovasculares, reforçando os achados de pesquisas anteriores sobre a efetividade de estratégias de estímulo ao autocuidado em pessoas com DM2^22^ ^-^ ^24^. 

A abordagem individualizada, com pactuação de metas realistas e acompanhamento contínuo, favoreceu o envolvimento ativo dos participantes no cuidado. Esse processo colaborativo, pautado na escuta e na corresponsabilidade estimula o protagonismo e a autoconfiança das pessoas com DM2, corroborando estudos que destacam o papel do enfermeiro como facilitador de mudanças comportamentais sustentáveis^3^ ^,^ ^9^ ^,^ ^25^.

O telemonitoramento mostrou-se um recurso importante de suporte entre as consultas, possibilitando o reforço das orientações, a revisão de metas e o esclarecimento de dúvidas. Essa modalidade de acompanhamento amplia o acesso, fortalece o vínculo terapêutico e sustenta comportamentos de autocuidado ao longo do tempo. Estes benefícios também foram descritos em estudo que utilizou um aplicativo de saúde móvel como forma de intervenção^26^.

Além dos benefícios clínicos e comportamentais, os achados do presente estudo reforçam a relevância do cuidado centrado no paciente com valorização do contexto social, cultural e emocional de cada pessoa. Destarte, o uso de linguagem acessível, a construção compartilhada do plano de cuidados e o incentivo à autonomia são estratégias que tornam o processo educativo mais significativo e eficaz^27^ ^-^ ^28^.

A redução significativa nos níveis de colesterol total, VLDL e triglicérides nos participantes do grupo intervenção pode estar relacionada às principais metas pactuadas durante as CE que foram a melhora nas práticas de alimentação e atividade física. Essa evolução reflete o impacto positivo da intervenção no conhecimento, na autonomia e no engajamento dos participantes, aspectos centrais no manejo de condições crônicas^9^ ^,^ ^23^. 

No entanto, os resultados divergem de uma revisão sistemática de 18 estudos conduzidos na África, totalizando 2.599 participantes, que não evidenciou melhorias estatisticamente significativas no perfil lipídico após intervenções voltadas ao autogerenciamento do diabetes^29^. Os autores atribuíram tais achados a limitações referentes e à heterogeneidade das intervenções, bem como à necessidade de profissionais capacitados, recursos adequados e atenção à alfabetização em saúde das pessoas com DM2.

Na perspectiva do manejo clínico, o efeito observado sobre o colesterol total difere de estudo realizado na Cidade do México, junto a 2.334 pessoas, em que se observou aumento discreto do colesterol total como efeito da aplicação de ferramenta multimídia^30^. Acerca do efeito sobre os triglicérides, o mesmo estudo encontrou redução de 7,6% (de 227,78 para 210,38 mg/dl; p = 0,001), o que sinaliza que a integração entre estratégias (consulta de enfermagem com telemonitoramento) pode favorecer o alcance de melhores resultados.

O erro-padrão do coeficiente estimado para a glicemia de jejum foi considerado alto (57,5% do valor do coeficiente), o que pode estar diretamente relacionado ao fato de a diferença entre os grupos não ter sido considerada estatisticamente significativa. Contudo, o efeito observado sobre a glicemia de jejum é comparável aos efeitos observados na literatura e que foram considerados significativos também do ponto de vista estatístico^31^. 

Em uma intervenção realizada por enfermeiros, baseada em consultas de enfermagem e em plano de cuidados junto a 249 pessoas com DM2 na Espanha, encontrou-se redução de 10,1% nos valores glicêmicos (baseados na HbA1c)^31^. Já em outro estudo realizado na Tailândia, o efeito ajustado observado foi de uma redução de 7,3% nos valores glicêmicos em 77 pessoas com DM2, a partir de uma intervenção durante 4 meses baseada em educação para o autogerenciamento do diabetes^32^.

A incorporação de práticas baseadas no autocuidado apoiado na APS é viável e coerente com os princípios da integralidade e da continuidade do cuidado. Contudo, a disposição do profissional não basta, é preciso investimento em capacitação profissional, reorganização dos processos de trabalho e apoio institucional para garantir a sustentabilidade das ações e a integração entre os diferentes níveis de atenção^33^. O estudo realizado no sul do Brasil constatou que apesar de os enfermeiros acreditarem que o uso das tecnologias pode favorecer a comunicação com outros pontos da RAS e com o paciente, otimizar e apoiar o trabalho em equipe, eles consideram que ausência de capacitações e de apoio institucional e insuficiência de equipamentos e recursos humanos são fatores que afetam e podem inviabilizar o uso do telemonitoramento no acompanhamento de pessoas com condições crônicas como hipertensão e diabetes na APS^34^. 

A reorganização dos processos de trabalho é um requisito para que as práticas que endossem o engajamento dos usuários em condições crônicas se deem sob o escopo proposto no Modelo de Atenção às Condições Crônicas. A implementação da política pública de saúde passa por entraves no microcenário da atenção à saúde e que, no caso do cuidado em condições crônicas, envolve: divergências entre o cumprimento de tarefas individuais dos profissionais e o envolvimento coletivo, predominância do atendimento curativo, falta de planejamento e de integração profissional e a pressão de trabalho relacionada a metas e prazos baseados na produtividade^35^.

Observa-se na literatura que a oferta de suporte técnico-pedagógico aos profissionais da APS faz parte do processo de reorganização da atenção e que o mesmo contribuiu para a implementação de tecnologias leves e leves-duras no âmbito desse nível assistencial e, consequentemente, para a estabilização do estado de saúde dos usuários com condições crônicas^36^. Destaca-se, portanto, que para a aplicação prática do autocuidado apoiado na APS, o passo inicial deve envolver gestão, setores de educação permanente e universidades no suporte aos profissionais do ponto de atenção à saúde, a fim de que se sintam apoiados e envolvidos pela cultura da corresponsabilização entre profissional-usuário. 

Embora o presente estudo demonstre que o uso dos princípios do autocuidado apoiado pelo enfermeiro produza efeitos positivos sobre os resultados clínicos, entende-se que sua aplicabilidade ultrapassa a prática uniprofissional. Para a realidade nacional, o envolvimento dos ACS é condição fundamental ao considerar a implantação e sustentação das práticas de engajamento, aconselhamento e telemonitoramento a tais condições^37^. O papel de médicos, profissionais de educação física, nutricionistas e psicólogos deve ser considerado conforme as especificidades locorregionais da RAS e as necessidades e metas dos usuários. Oportunizar as modalidades individual e de Cuidado Compartilhado em grupo podem ampliar o alcance do autocuidado apoiado na prática^24^ ^-^ ^25^. 

Dentre as possíveis limitações deste estudo, destaca-se a ausência da estratificação do grau de risco, além de fatores externos, como falhas na distribuição de medicamentos durante o período de coleta de dados, que não foram controlados na análise dos dados. Outro aspecto a ser considerado foi a dificuldade em obter o envolvimento ativo dos enfermeiros da ESF nas CE como acordado inicialmente, uma vez que a proposta visava a aplicabilidade no SUS. As justificativas apresentadas para a baixa adesão incluíram o cotidiano sobrecarregado da atenção básica e a percepção de que a presença da pesquisadora principal na condução das atividades dispensaria a participação direta dos demais profissionais. De qualquer modo, os resultados oferecem subsídios para o fortalecimento da enfermagem como prática científica e social, evidenciando o potencial da CE baseada no autocuidado apoiado para promover a autonomia dos indivíduos no manejo do diabetes e melhorar desfechos clínicos.

## Conclusão

A consulta de enfermagem baseada no autocuidado apoiado demonstrou efeito positivo no engajamento das práticas de autocuidado em pessoas com DM2 acompanhadas na APS e sobre parâmetros laboratoriais, especialmente colesterol total, lipoproteína de muito baixa densidade e triglicérides. Esses resultados evidenciam que intervenções educativas estruturadas, centradas na pactuação de metas e acompanhadas de suporte contínuo, podem promover o engajamento, autonomia e melhor manejo da condição crônica.

Os achados reforçam o papel estratégico do enfermeiro na implementação de práticas inovadoras e humanizadas de cuidado, capazes de integrar aspectos clínicos, educativos e relacionais. A adoção sistemática da CE fundamentada no autocuidado apoiado contribui para a consolidação de um modelo assistencial centrado no paciente e alinhado aos princípios da integralidade e da corresponsabilização.

Recomenda-se que estudos futuros ampliem as estratégias de intervenção, incluindo a análise dos efeitos da atuação multiprofissional, de abordagens coletivas, de tecnologias digitais e de diferentes contextos assistenciais. Essa ampliação de abordagens poderá fortalecer as evidências sobre a efetividade e aplicabilidade do autocuidado apoiado no enfrentamento das condições crônicas.

## Data Availability

Os conjuntos de dados relacionados a este artigo estarão disponíveis mediante solicitação ao autor correspondente.

# Translation: Consulta de Enfermería basada en el autocuidado apoyado para el manejo de la diabetes mellitus tipo 2: estudio cuasi experimental^*^

**DOI:** 10.1590/1518-8345.8132.4872

**Authors:** Elen Ferraz Teston, Gabrielly Segatto Brito, Rosilene Rocha Palasson, Thaís Gianini Dias, Guilherme Oliveira de Arruda, Sonia Silva Marcon

**Affiliations:** 1 Universidade Federal de Mato Grosso do Sul, Instituto Integrado de Saúde, Campo Grande, MS, Brasil.; 2 Scholarship holder at the Coordenação de Aperfeiçoamento de Pessoal de Nível Superior (CAPES), Brasil.; 3 Universidade Estadual de Maringá, Departamento de Enfermagem, Maringá, PR, Brasil.

**Keywords:** Enfermería de Consulta, Diabetes Mellitus, Autocuidado, Participación del Paciente, Atención Primaria de Salud, Enfermería

## Abstract

**Objetivo:**

evaluar el efecto de la consulta de enfermería basada en el autocuidado apoyado en la realización de actividades de autocuidado y sobre parámetros de laboratorio en personas con diabetes mellitus tipo 2.

**Método:**

estudio cuasi experimental con personas con diabetes mellitus tipo 2, con grupo comparador, cuyos participantes recibieron el seguimiento habitual ofrecido por la unidad, y grupo intervención, que recibió tres consultas de enfermería y dos llamadas telefónicas de monitoreo. Los datos se analizaron mediante modelos lineales mixtos, con nivel de significación del 5%.

**Resultados:**

participaron en el estudio 57 individuos. Se observó un efecto significativo de la intervención sobre los puntajes de adherencia a las actividades de autocuidado y en los niveles de colesterol total, lipoproteína de muy baja densidad, triglicéridos.

**Conclusión:**

la consulta de enfermería centrada en el apoyo al autocuidado asociada al monitoreo telefónico favorece la comprensión sobre la enfermedad y el compromiso con el manejo de la condición crónica por parte de personas con diabetes mellitus tipo 2, con repercusiones en los parámetros de laboratorio.

## Destacados

**(1)** Aumento de la adherencia a las prácticas de autocuidado y mejora de los parámetros de laboratorio. **(2)** Autocuidado apoyado en la APS: práctica viable, sostenible y centrada en el paciente. **(3)** Papel estratégico del enfermero en la implementación de prácticas innovadoras y humanizadas. **(4)** Contribución del abordaje telefónico en el apoyo continuo al cuidado. **(5)** Modelo sostenible en la APS amplía el compromiso y la eficiencia en el manejo de la DM2.

## Introducción

La consulta de enfermería (CE) exige razonamiento clínico e integra evaluaciones sistemáticas, intervenciones individualizadas y acciones educativas orientadas a la promoción del cuidado integral. Las orientaciones realizadas durante las CE se adaptan a las necesidades y singularidades de los usuarios y de sus familias^1^, considerándose una tecnología blanda-dura, sostenida en saberes estructurados y fundamentos técnico-científicos que orientan el proceso de trabajo del enfermero^2^.

Las acciones educativas orientadas a la promoción de la salud son esenciales, pues estimulan a las personas a reflexionar sobre sus comportamientos y necesidades de cambios que favorezcan el bienestar y el manejo de condiciones crónicas. Estudios indican que intervenciones basadas en el autocuidado apoyado ejercen un impacto positivo en la autogestión de la salud de personas con condición crónica^3^ ^-^ ^5^, como la diabetes mellitus tipo 2 (DM2). El autocuidado apoyado se fundamenta en la metodología de las 5A (Analizar, Aconsejar, Acordar, Asistir y Acompañar), que organiza de forma estructurada el proceso de apoyo al cambio de comportamiento, orientando al profesional en la evaluación de las necesidades del usuario, en el asesoramiento cualificado, en el establecimiento de metas de manera pactada y en el seguimiento sistemático^6^.

De manera más amplia, el autocuidado constituye un componente esencial en la promoción de la salud y en el manejo de las condiciones crónicas, entendiéndose como un proceso multidimensional que involucra conocimiento, desarrollo de habilidades y cambio de actitud para el monitoreo y el mantenimiento de la propia condición de salud^5^ ^,^ ^7^ ^-^ ^8^. Cuando se apoya en una comunicación efectiva y centrada en la perspectiva del usuario, este proceso contribuye al fortalecimiento de la confianza y de la corresponsabilización de las personas en relación con su propio cuidado^8^ ^-^ ^9^.

La *American Diabetes Association* (ADA) recomienda firmemente intervenciones en el estilo de vida como estrategia central para el manejo de la DM2^10^. En este manejo, el autocuidado es esencial e involucra actividades como el monitoreo frecuente de la glucemia capilar, alimentación saludable, práctica de actividad física regular, uso correcto de las medicaciones y cuidados con los pies^11^.

En la Atención Primaria de Salud (APS), la CE fundamentada en el marco del autocuidado apoyado puede constituir una estrategia prometedora para promover el compromiso de las personas con relación a su condición de salud. Por medio de la evaluación clínica, la pactación de metas, la elaboración de planes de cuidado individualizados y el seguimiento continuo, el enfermero puede ayudar a las personas con DM2 a realizar un manejo más eficaz de su condición de salud, utilizando los recursos disponibles en los servicios y en la comunidad^1^ ^,^ ^6^ ^,^ ^12^.

Aunque los estudios señalan la importancia de las intervenciones centradas en el paciente para la promoción de la salud y la autogestión de la condición crónica, se constata el predominio de aquellos enfocados en la prevención de complicaciones de la enfermedad^13^ ^-^ ^15^. Por lo tanto, persisten lagunas en la literatura que traduzcan las recomendaciones en prácticas estructuradas de enfermería en el ámbito de la APS^16^.

En este sentido, evidenciar que el uso de una tecnología blanda-dura como la CE puede favorecer el manejo adecuado de la DM2 y, a la vez, contribuir al proceso de trabajo en las unidades de Salud de la Familia, a la reducción de hospitalizaciones, a la adherencia al tratamiento y a las prácticas de autocuidado, y a la mejora de la calidad de vida^1^ ^,^ ^3^ ^,^ ^10^. Además, también contribuye al avance del conocimiento sobre la atención ofrecida a las personas con condiciones crónicas, especialmente la diabetes.

Ante lo expuesto, surge el siguiente interrogante: ¿Cuáles son los efectos de la CE basada en el autocuidado apoyado en la adopción de prácticas de autocuidado y sobre los parámetros de laboratorio de personas con DM2? Para responderlo, se definió como objetivo del estudio: evaluar el efecto de la CE basada en el autocuidado apoyado en la realización de actividad de autocuidado y sobre parámetros de laboratorio en personas con DM2.

## Método

### Diseño del estudio

Estudio cuasi experimental, de naturaleza cuantitativa, vinculado a un proyecto matriz financiado por la *Fundação de Apoio ao Desenvolvimento do Ensino Ciência e Tecnologia do Estado de Mato Grosso do Sul* (FUNDECT), en la convocatoria del *Programa Pesquisa para o SUS: gestão compartilhada em saúde* (PPSUS). Las recomendaciones del *Transparent Reporting of Evaluations With Nonrandomized Designs* (TREND)^17^ se utilizaron en la elaboración de este informe de investigación.

### Lugar y período de la recolección de datos

El estudio se realizó en dos Unidades de Salud de la Familia (USFs) en una capital del Centro-Oeste brasileño. La primera tenía 447 personas con DM2 registradas y fue destinada al grupo intervención; la segunda tenía 332 personas registradas y fue destinada al grupo comparador. Los datos se recolectaron en el período de marzo a noviembre de 2023.

### Población y criterios de selección

La población del estudio estuvo compuesta por personas con DM2. Los criterios de inclusión fueron: 18 años o más, estar registrado en una de las USF en estudio y manifestar interés en participar en la investigación al tener conocimiento de la misma. Los criterios de exclusión fueron: presentar condiciones de salud que dificultaran o imposibilitaran la comunicación verbal y/o el desplazamiento hasta la unidad, según lo informado por el ACS.

Se consideraron como motivos de discontinuidad: hospitalización en el período de recolección de datos y la no atención de tres intentos de contacto telefónico en días y horarios distintos. 

### Definición de la muestra y variables de estudio

La investigación se difundió mediante carteles fijados en las unidades, distribución de folletos e invitaciones personales realizadas por los miembros del equipo de Salud de la Familia, sobre todo los Agentes Comunitarios de Salud (ACS). La muestra fue de tipo no probabilístico, definida por conveniencia.

Las variables de interés fueron aquellas relacionadas con la caracterización de los participantes (grupo etario, sexo, raza, estado civil, ocupación, escolaridad, tiempo de diagnóstico) y con los desenlaces. Se consideraron dos tipos específicos de desenlaces: a) Parámetros de laboratorio de los exámenes: glucemia en ayunas, colesterol total, lipoproteína de alta densidad (HDL), lipoproteína de baja densidad (LDL), lipoproteína de muy baja densidad (VLDL), triglicéridos, urea, creatinina, hemoglobina glucosilada (HbA1c); y b). Actividades de autocuidado para la diabetes.

### Caracterización de la intervención

Los individuos participantes del grupo comparador recibieron el seguimiento habitual de la unidad a la que estaban vinculados, que incluye consultas médicas a demanda, distribución de medicamentos y de insumos para la aplicación de insulina y el monitoreo de la glucemia. Para la investigación se realizaron exámenes de laboratorio y respondieron un cuestionario en dos momentos. Ya los integrantes del grupo intervención, además de lo ya citado, recibieron también tres CE presenciales y dos llamadas telefónicas de monitoreo.

Las CE fueron guiadas por un instrumento, elaborado por el equipo de investigación con base en la información contenida en el manual de Autocuidado, el cual se fundamenta en la metodología de las “5 A” (Analizar, Aconsejar, Acordar, Asistir y Acompañar)^18^. Tuvieron una duración media de 60 minutos y se realizaron conforme al cronograma presentado en la Figura 1, en la propia unidad o en el domicilio, cuando fue estrictamente necesario, debido a una intercurrencia que imposibilitó el desplazamiento del paciente (dos casos).

**Figura 1:** Esquema de intervención

En la primera CE se realizó el examen físico y se investigó la percepción sobre la diabetes, además de identificar las principales dificultades/problemas enfrentados para el manejo de esta condición. También se realizó una evaluación clínica compuesta por toma de la presión arterial, verificación del peso y la talla para la determinación del Índice de Masa Corporal, circunferencia abdominal y medición de la glucemia capilar.

Al final de la CE se solicitó que los participantes priorizaran uno de los problemas/dificultades en el manejo de la DM2 y sugirieran acciones que consideraban viables para afrontarlo. A partir de ello, se elaboró, de forma conjunta, un plan de cuidados individualizado para cada participante, constituido por metas y actividades a realizar.

En las dos consultas siguientes, la evaluación clínica se realizó nuevamente y se investigó el cumplimiento de las metas. Cuando las metas se alcanzaban, el individuo señalaba otro problema/dificultad y sugería acciones para afrontarlo(a), estableciendo una nueva meta. Si la meta no se alcanzaba en el período de treinta días, se discutía nuevamente, con base en los deslices y recaídas del proceso de cambio de comportamiento, y se repactaba, respetando los límites y las posibilidades del individuo.

El telemonitoreo, a su vez, tuvo como objetivo acompañar la meta pactada, identificar las expectativas para la próxima consulta, aclarar dudas durante el proceso de cambio de hábito y ayudar a los individuos en las dificultades relacionadas con el seguimiento de las metas. Para cada participante, se creó un historial clínico físico que contenía los registros de la información obtenida durante las CE y los telemonitoreos.

La investigadora principal (enfermera, maestranda en Enfermería, debidamente capacitada para la realización de la CE, la elaboración de planes de cuidados y la aplicación de los instrumentos de recolección de datos), que no tenía ningún tipo de relación previa con los participantes, fue responsable de la realización de todas las CE y los telemonitoreos.

### Recolección de datos e instrumento utilizado

En los períodos pre y posintervención, los participantes de ambos grupos se realizaron exámenes de laboratorio de rutina para el seguimiento de la DM2 y respondieron el Cuestionario de Actividades de Autocuidado con la Diabetes (CAD). Los resultados de los exámenes se imprimieron en dos copias: una entregada al paciente y otra archivada en la historia clínica.

Para evaluar la adherencia a las actividades de autocuidado se utilizó el CAD, traducido, adaptado y validado para Brasil^19^. El CAD contiene 15 ítems que abordan alimentación, actividad física, monitoreo de la glucemia, cuidado de los pies, uso de medicación y tabaquismo. Las respuestas se presentan en una escala tipo Likert (cero a siete), correspondiente al número de días de la semana en que se realizó la actividad, siendo cero la peor y siete la mejor situación posible. La puntuación se invierte en los ítems sobre el consumo de grasas y dulces^20^. Al inicio de la investigación, el CAD fue aplicado por la investigadora principal junto a los integrantes de los dos grupos, cuando estos acudían a la Unidad para la recolección de material para los exámenes de laboratorio. 

### Tratamiento y análisis de los datos

Los datos se tabularon en una hoja de cálculo del *software* Excel. Los registros de las CE y de los telemonitoreos se analizaron con el propósito de cuantificar las metas de autocuidado pactadas con mayor frecuencia. Se emplearon las pruebas t para muestras independientes, X^2^ de Pearson y Exacto de Fisher para la comparación de los grupos de estudio en la línea de base, según características sociodemográficas y tiempo de diagnóstico. Se realizó la prueba de Wilcoxon para la comparación no ajustada de los puntajes de los grupos entre los dos momentos de observación.

Se realizó un análisis mediante modelos lineales mixtos (*Linear Mixed Models* - LMM) para evaluar el efecto de la intervención sobre el autocuidado a lo largo del tiempo y sobre los valores de los exámenes de laboratorio. Este enfoque se eligió por considerar la dependencia entre las mediciones repetidas realizadas en los mismos participantes y por permitir la inclusión de casos con datos ausentes en uno o más momentos de observación, bajo el supuesto de que los datos faltantes eran del tipo aleatorio (*Missing at Random* - MAR)^21^.

El momento pre y posintervención se especificó como factor intra-sujeto (efecto de medidas repetidas) y el grupo (intervención vs comparador) como factor inter-sujetos. Se incluyó el efecto de interacción “momento × grupo” para probar si la variación del desenlace a lo largo del tiempo difirió entre los grupos, representando el efecto de la intervención.

Los modelos se ajustaron con intercepto aleatorio por participante, lo que permitió estimar la variabilidad individual en los valores de línea de base. Se adoptó la estructura de covarianza “*compound symmetry*”, considerando la correlación constante entre las medidas repetidas (adecuada para dos momentos de observación). Los análisis se ajustaron por covariables potencialmente confundidoras previamente definidas (edad, sexo, raza/color y tiempo de diagnóstico), incluidas como efectos fijos.

Los supuestos de normalidad y homocedasticidad de los residuos se verificaron gráficamente (histograma, *Q-Q plot* y gráfico de residuos estandarizados versus valores predichos). Se consideraron tolerables pequeñas violaciones, dado el carácter robusto de los modelos mixtos. Se adoptó el nivel de significancia del 5%. Los análisis se realizaron en el *software* IBM *Statistical Package for the Social Sciences* (SPSS)^®^ *Statistics*, versión 20.

### Consideraciones éticas

El estudio se desarrolló en conformidad con las recomendaciones éticas nacionales e internacionales para investigaciones que involucran seres humanos, con aprobación por el Comité de Ética en Investigación con Seres Humanos de la institución signataria (Dictamen n.º 4.321.389).

## Resultados

Se asignaron 29 individuos al grupo intervención y 28 al grupo comparador. La Figura 2 presenta el flujo del estudio desde la inclusión hasta el análisis de los datos.

**Figura 2:** Diagrama de flujo de inclusión, asignación, seguimiento y análisis de los participantes del estudio

*USF = Unidad de Salud de la Familia

Los participantes de ambos grupos se consideraron semejantes desde el punto de vista estadístico. Aunque el resultado de la prueba de comparación de proporciones indique diferencia significativa para la escolaridad, se observa que los intervalos de confianza se superponen en alguna medida para todas las proporciones observadas (Tabla 1).

**Tabla 1:** Comparación de los grupos Intervención y Comparador con base en características sociodemográficas y tiempo de diagnóstico en la línea de base. Campo Grande, MS, Brasil 2023-2024

Variables sociodemográficas	Grupo Intervención		Grupo Comparador		p-valor
	N (%)* o Media (De)^‡^	IC-95%^†^	N (%)* o Media (De)^‡^	IC-95%^†^	
**Edad (años)**	64,7 (11,3)	60,4 - 69,0	66,2 (8,64)	62,6 - 69,8	0,597^§^
**Sexo**					
Femenino	20 (69,0)	50,6 - 82,8	20 (76,9)	57,5 - 89,2	0,508^||^
Masculino	9 (31,0)	17,2 - 49,4	6 (23,1)	10,8 - 42,5	
**Raza**					
Blanco	15 (51,7)	34,4 - 68,6	12 (46,2)	28,8 - 64,5	0,187^¶^
Pardo	6 (20,7)	9,6 - 38,9	10 (38,5)	22,4 - 57,5	
Negro	8 (27,6)	14,6 - 46,0	3 (11,5)	3,33 - 30,0	
Amarillo	0 (0,0)	0,00 - 0,00	1 (3,8)	0,00 - 20,7	
**Estado civil**					
Casado	18 (62,1)	43,9 - 77,3	11 (44,0)	26,7 - 62,9	0,342^¶^
Soltero	2 (6,9)	0,09 - 23,3	5 (20,0)	8,5 - 39,7	
Separado	2 (6,9)	0,09 - 23,3	0 (0,0)	0,00 - 16,1	0,342^¶^
Divorciado	3 (10,3)	2,9 - 27,4	4 (16,0)	5,9 - 35,4	
Viudo	4 (13,8)	5,0 - 31,3	5 (20,0)	8,5 - 39,7	
**Ocupación**					
Jubilado	13 (44,8)	28,4 - 62,4	11 (42,3)	25,6 - 61,1	0,262^||^
Ama de casa	6 (20,7)	9,6 - 38,9	10 (38,5)	22,4 - 57,5	
Otros	10 (34,5)	19,9 - 52,8	5 (19,2)	8,2 - 38,5	
**Nivel Educativo**					
Analfabeto	2 (6,9)	0,09 - 23,3	1 (3,8)	0,00 - 20,7	0,031^¶^
Primaria Incompleta	12 (41,4)	25,5 - 59,3	20 (76,9)	57,5 - 89,2	
Primaria Completa	9 (31,0)	17,2 - 49,4	1 (3,8)	0,00 - 20,7	
Secundaria Incompleta	1 (3,4)	0,00 - 18,9	1 (3,8)	0,00 - 20,7	
Secundaria Completa	4 (13,8)	5,0 - 31,3	3 (11,5)	3,3 - 30,0	
Educación Superior Completa	1 (3,4)	0,00 - 18,9	0 (0,0)	0,00 - 15,6	
**Tiempo de diagnóstico (años)**	7,31 (7,58)	3,9 - 10,6	10,3 (10,3)	6,6 - 13,9	0,234^§^

*N (%) = Número absoluto y porcentaje; ^†^IC-95% = Intervalo de confianza para la proporción; ^‡^De = Desviación Estándar; ^§^Prueba t para muestras independientes; ^||^X^2^ de Pearson; ^¶^Prueba Exacta de Fisher

La meta pactada con mayor frecuencia durante las CE fue la alimentación saludable (65,52%), con énfasis en la reducción de grasas y carbohidratos y un mayor consumo de frutas. La práctica de actividad física, principalmente caminatas tres veces por semana, durante 30 minutos, fue la segunda meta más pactada (58,62%), seguida por el aumento en la ingesta de agua (37,93%) y el manejo del estrés mediante actividades placenteras (27,59%).

De acuerdo con la Tabla 2, los puntajes de actividades de autocuidado presentaron diferencia significativa entre los momentos pre y pos para el grupo intervención, mientras que, para el grupo comparador, la diferencia no fue estadísticamente significativa.

**Tabla 2:** Comparación de los puntajes de Actividades de Autocuidado con la Diabetes en el Grupo Intervención y el Grupo Comparador en los momentos pre y posintervención. Campo Grande, MS, Brasil, 2023-2024

Puntajes	Momentos						p-valor*
	Preintervención			Posintervención			
	Media	Mediana	Desviación estándar	Media	Mediana	Desviación estándar	
Grupo Intervención CAD^†^	3,57	3,43	0,82	4,29	4,43	0,76	<0,001
Grupo Comparador CAD^†^	3,65	3,57	0,92	3,98	3,72	1,20	0,084

*Prueba de Wilcoxon; ^†^CAD = Cuestionario de Actividades de Autocuidado con la Diabetes

El modelo lineal mixto indicó un efecto significativo de la interacción “momento × grupo” sobre el desenlace (F (50,0)=5,30; p=0,025), demostrando que la intervención produjo un efecto significativamente distinto del resultado obtenido por el grupo comparador. El efecto superior de la intervención se hace evidente al analizar el coeficiente estimado y ajustado por las variables sexo, edad, raza/color y tiempo de diagnóstico, que fue igual a 0,55 (error estándar = 0,23; IC95% = 0,07 - 1,03). Por lo tanto, la variación positiva en los valores del puntaje de autocuidado con la diabetes entre las personas que recibieron la intervención fue significativamente diferente de la variación en el grupo comparador a lo largo del tiempo.

La Tabla 3 muestra que, de acuerdo con los modelos lineales mixtos, hubo un efecto positivo significativo de la intervención sobre los valores de colesterol total, VLDL y triglicéridos. Esto evidencia que la variación entre los momentos pre y posintervención fue diferente entre los grupos independientemente de las covariables analizadas.

**Tabla 3:** Efectos de la intervención con base en la interacción “momento × grupo” sobre los valores de exámenes de laboratorio. Campo Grande, MS, Brasil, 2023-2024

Variable	Coeficiente (β)*	Error estándar	IC95%^†^ Inferior	IC95%^†^ Superior	p-valor^‡^
Glucemia en ayunas	- 28,59	16,44	61,62	4,43	0,088
Colesterol Total	23,23	8,86	41,05	5,42	**0,012**
HDL^‡^	0,12	1,89	3,93	3,69	0,949
LDL^§^	0,08	6,74	13,47	13,65	0,990
VLDL^||^	8,74	2,80	14,37	3,11	**0,003**
Triglicéridos	73,18	26,84	127,11	19,26	**0,009**
Urea	4,10	2,57	9,27	1,07	0,118
Creatinina	0,02	0,03	0,09	0,04	0,480
HbA1c^¶^	0,57	0,37	1,31	0,17	0,130

*β = Coeficiente estimado, ajustado por las variables sexo, edad, raza/color y tiempo de diagnóstico; ^†^IC95% = Intervalo de confianza del 95%; ^‡^Modelos lineales mixtos; ^‡^HDL = Lipoproteína de alta densidad; ^§^LDL = Lipoproteína de baja densidad; ^||^VLDL = Lipoproteína de muy baja densidad; ^¶^HbA1c = Hemoglobina glucosilada. El término de interacción Momento×Grupo representa el efecto diferencial de la intervención entre los grupos a lo largo del tiempo

En términos proporcionales y considerando los valores del coeficiente estimado contenidos en la Tabla 3 (que descuenta el efecto sobre el grupo comparador), se destacan las reducciones en los valores de glucemia en ayunas, colesterol total y triglicéridos para el Grupo Intervención, que fueron, respectivamente, del 16,7% (media = 171,41 mg/dl), 11,7% (media = 199,52 mg/dl) y 32% (media = 228,55 mg/dl).

## Discusión

Los resultados de este estudio demostraron efectos positivos de la CE basada en el autocuidado apoyado sobre las prácticas de autocuidado y los niveles de colesterol total, lipoproteína de muy baja densidad y triglicéridos de personas con DM2 seguidas en la APS. Este resultado indica que intervenciones no farmacológicas conducidas por enfermeros pueden contribuir al manejo de la enfermedad y a la prevención de complicaciones cardiovasculares, reforzando los hallazgos de investigaciones anteriores sobre la efectividad de estrategias de estímulo al autocuidado en personas con DM2^22^ ^-^ ^24^.

El abordaje individualizado, con pactación de metas realistas y seguimiento continuo, favoreció la participación activa de los participantes en el cuidado. Este proceso colaborativo, basado en la escucha y en la corresponsabilidad, estimula el protagonismo y la autoconfianza de las personas con DM2, corroborando estudios que destacan el papel del enfermero como facilitador de cambios conductuales sostenibles^3^ ^,^ ^9^ ^,^ ^25^.

El telemonitoreo se mostró como un recurso importante de apoyo entre las consultas, posibilitando el refuerzo de las orientaciones, la revisión de metas y la aclaración de dudas. Esta modalidad de seguimiento amplía el acceso, fortalece el vínculo terapéutico y sostiene conductas de autocuidado a lo largo del tiempo. Estos beneficios también fueron descritos en un estudio que utilizó una aplicación de salud móvil como forma de intervención^26^.

Además de los beneficios clínicos y conductuales, los hallazgos del presente estudio refuerzan la relevancia del cuidado centrado en el paciente, con valorización del contexto social, cultural y emocional de cada persona. Por lo tanto, el uso de un lenguaje accesible, la construcción compartida del plan de cuidados y el incentivo a la autonomía son estrategias que hacen que el proceso educativo sea más significativo y eficaz^27^ ^-^ ^28^.

La reducción significativa en los niveles de colesterol total, VLDL y triglicéridos en los participantes del grupo intervención puede estar relacionada con las principales metas pactadas durante las CE, que fueron la mejora de las prácticas de alimentación y actividad física. Esta evolución refleja el impacto positivo de la intervención en el conocimiento, la autonomía y el compromiso de los participantes, aspectos centrales en el manejo de condiciones crónicas^9^ ^,^ ^23^.

Sin embargo, estos resultados difieren de una revisión sistemática de 18 estudios realizados en África, que totalizó 2.599 participantes, la cual no evidenció mejoras estadísticamente significativas en el perfil lipídico tras intervenciones orientadas a la autogestión de la diabetes^29^. Los autores atribuyeron tales hallazgos a limitaciones y a la heterogeneidad de las intervenciones, así como a la necesidad de profesionales capacitados, recursos adecuados y atención a la alfabetización en salud de las personas con DM2.

Desde la perspectiva del manejo clínico, el efecto observado sobre el colesterol total difiere de un estudio realizado en la Ciudad de México, con 2.334 personas, en el que se observó un aumento discreto del colesterol total como efecto de la aplicación de una herramienta multimedia^30^. En cuanto al efecto sobre los triglicéridos, el mismo estudio encontró una reducción del 7,6% (de 227,78 a 210,38 mg/dl; p = 0,001), lo que indica que la integración entre estrategias (consulta de enfermería con telemonitoreo) puede favorecer el logro de mejores resultados.

El error estándar del coeficiente estimado para la glucemia en ayunas se consideró alto (57,5% del valor del coeficiente), lo que puede estar directamente relacionado con el hecho de que la diferencia entre los grupos no se consideró estadísticamente significativa. No obstante, el efecto observado sobre la glucemia en ayunas es comparable a efectos observados en la literatura y que también se consideraron significativos desde el punto de vista estadístico^31^.

En una intervención realizada por enfermeros, basada en consultas de enfermería y en un plan de cuidados, con 249 personas con DM2 en España, se encontró una reducción del 10,1% en los valores glucémicos (basados en la HbA1c)^31^. Ya en un estudio realizado en Tailandia, el efecto ajustado observado fue una reducción del 7,3% en los valores glucémicos en 77 personas con DM2, a partir de una intervención durante 4 meses basada en educación para la autogestión de la diabetes^32^.

La incorporación de prácticas basadas en el autocuidado apoyado en la APS es viable y coherente con los principios de la integralidad y de la continuidad del cuidado. Sin embargo, no basta la disposición del profesional; requiere inversión en capacitación profesional, reorganización de los procesos de trabajo y apoyo institucional para garantizar la sostenibilidad de las acciones y la integración entre los diferentes niveles de atención^33^. Un estudio realizado en el sur de Brasil constató que, aunque los enfermeros creen que el uso de tecnologías puede favorecer la comunicación con otros puntos de la RAS y con el paciente, optimizar y apoyar el trabajo en equipo, consideran que la ausencia de capacitaciones y de apoyo institucional, y la insuficiencia de equipos y recursos humanos, son factores que afectan y pueden inviabilizar el uso del telemonitoreo en el seguimiento de personas con condiciones crónicas como hipertensión y diabetes en la APS^34^. 

La reorganización de los procesos de trabajo es un requisito para que las prácticas que respalden el compromiso de los usuarios con condiciones crónicas se desarrollen bajo el alcance propuesto en el Modelo de Atención a las Condiciones Crónicas. La implementación de la política pública de salud enfrenta obstáculos en el microescenario de la atención sanitaria y que, en el caso del cuidado de condiciones crónicas, involucra: divergencias entre el cumplimiento de tareas individuales de los profesionales y la participación colectiva, predominio de la atención curativa, falta de planificación y de integración profesional, y la presión laboral relacionada con metas y plazos basados en la productividad^35^.

Se observa en la literatura que la oferta de soporte técnico-pedagógico a los profesionales de la APS forma parte del proceso de reorganización de la atención y que este contribuyó a la implementación de tecnologías blandas y blando-duras en el ámbito de este nivel asistencial y, en consecuencia, a la estabilización del estado de salud de los usuarios con condiciones crónicas^36^. Se destaca, por lo tanto, que, para la aplicación práctica del autocuidado apoyado en la APS, el paso inicial debe involucrar a la gestión, a los sectores de educación permanente y a las universidades en el soporte a los profesionales del punto de atención a la salud, a fin de que estos se sientan apoyados e involucrados por la cultura de la corresponsabilización profesional-usuario. 

Aunque el presente estudio demuestra que el uso de los principios del autocuidado apoyado por el enfermero produce efectos positivos sobre los resultados clínicos, se entiende que su aplicabilidad trasciende la práctica uniprofesional. Para la realidad nacional, la participación de los ACS es condición fundamental al considerar la implantación y sustentación de las prácticas de compromiso, asesoramiento y telemonitoreo para tales condiciones^37^. El papel de médicos, profesionales de educación física, nutricionistas y psicólogos debe considerarse conforme a las especificidades locorregionales de la RAS y a las necesidades y metas de los usuarios. Favorecer las modalidades individuales y de Cuidado Compartido en grupo puede ampliar el alcance del autocuidado apoyado en la práctica^24^ ^-^ ^25^. 

Entre las posibles limitaciones de este estudio, se destacan la ausencia de estratificación del grado de riesgo, además de factores externos, como fallas en la distribución de medicamentos durante el período de recolección de datos, que no fueron controlados en el análisis de los datos. Otro aspecto a considerar fue la dificultad para lograr la participación activa de los enfermeros de la ESF en las CE, tal como se acordó inicialmente, dado que la propuesta apuntaba a la aplicabilidad en el SUS. Las justificaciones presentadas para la baja adherencia incluyeron el cotidiano sobrecargado de la atención básica y la percepción de que la presencia de la investigadora principal en la conducción de las actividades dispensaría la participación directa de los demás profesionales. De todos modos, los resultados ofrecen insumos para el fortalecimiento de la enfermería como práctica científica y social, evidenciando el potencial de la CE basada en el autocuidado apoyado para promover la autonomía de los individuos en el manejo de la diabetes y mejorar desenlaces clínicos.

## Conclusión

La consulta de enfermería basada en el autocuidado apoyado demostró un efecto positivo en el compromiso con las prácticas de autocuidado en personas con DM2 acompañadas en la APS y sobre parámetros de laboratorio, especialmente colesterol total, lipoproteína de muy baja densidad y triglicéridos. Estos resultados evidencian que intervenciones educativas estructuradas, centradas en la pactación de metas y acompañadas de soporte continuo, pueden promover el compromiso, la autonomía y un mejor manejo de la condición crónica.

Los hallazgos refuerzan el papel estratégico del enfermero en la implementación de prácticas innovadoras y humanizadas de cuidado, capaces de integrar aspectos clínicos, educativos y relacionales. La adopción sistemática de la CE fundamentada en el autocuidado apoyado contribuye a la consolidación de un modelo asistencial centrado en el paciente y alineado con los principios de la integralidad y de la corresponsabilización.

Se recomienda que estudios futuros amplíen las estrategias de intervención, incluyendo el análisis de los efectos de la actuación multiprofesional, de abordajes colectivos, de tecnologías digitales y de diferentes contextos asistenciales. Esta ampliación de abordajes podrá fortalecer las evidencias sobre la efectividad y aplicabilidad del autocuidado apoyado en el enfrentamiento de las condiciones crónicas.

## Data Availability

Los conjuntos de datos relacionados con este artículo estarán disponibles previa solicitud al autor correspondiente.
